# Angiotensin-(1-7) counteracts the transforming effects triggered by angiotensin II in breast cancer cells

**DOI:** 10.18632/oncotarget.19290

**Published:** 2017-07-17

**Authors:** Nadia Cambados, Thomas Walther, Karen Nahmod, Johanna M. Tocci, Natalia Rubinstein, Ilka Böhme, Marina Simian, Rocío Sampayo, Melisa Del Valle Suberbordes, Edith C. Kordon, Carolina Schere-Levy

**Affiliations:** ^1^ Instituto de Fisiología, Biología Molecular y Neurociencias, Facultad de Ciencias Exactas y Naturales, Universidad de Buenos Aires, Buenos Aires, Argentina; ^2^ Department of Obstetrics, University of Leipzig, Leipzig, Germany; ^3^ Department of Pediatric Surgery, University of Leipzig, Leipzig, Germany; ^4^ Department Pharmacology and Therapeutics, School of Medicine and School of Pharmacy, University College Cork, Cork, Ireland; ^5^ Institute of Medical Biochemistry and Molecular Biology, University Medicine Greifswald, Greifswald, Germany; ^6^ Department of Pediatrics, Immunology, Allergy and Rheumatology, Center for Human Immunobiology, Texas Children’s Hospital, Houston, Texas, USA; ^7^ Departamento de Fisiología, Biología Molecular y Celular, Facultad de Ciencias Exactas y Naturales, Universidad de Buenos Aires, Buenos Aires, Argentina; ^8^ Instituto de Nanosistemas, Universidad Nacional de San Martín, Buenos Aires, Argentina; ^9^ Departmento de Química Biológica, Facultad de Ciencias Exactas y Naturales, Universidad de Buenos Aires, Buenos Aires, Argentina

**Keywords:** AKT, angiotensin II, angiotensin-(1-7), breast cancer cells, epithelial–mesenchymal transition

## Abstract

Angiotensin (Ang) II, the main effector peptide of the renin-angiotensin system, has been implicated in multiple aspects of cancer progression such as proliferation, migration, invasion, angiogenesis and metastasis. Ang-(1-7), is a biologically active heptapeptide, generated predominantly from AngII by the enzymatic activity of angiotensin converting enzyme 2. Previous studies have shown that Ang-(1-7) counterbalances AngII actions in different pathophysiological settings. In this study, we have analysed the impact of Ang-(1-7) on AngII-induced pro-tumorigenic features on normal murine mammary epithelial cells NMuMG and breast cancer cells MDA-MB-231. AngII stimulated the activation of the survival factor AKT in NMuMG cells mainly through the AT1 receptor. This PI3K/AKT pathway activation also promoted epithelial–mesenchymal transition (EMT). Concomitant treatment of NMuMG cells with AngII and Ang-(1-7) completely abolished EMT features induced by AngII. Furthermore, Ang-(1-7) abrogated AngII induced migration and invasion of the MDA-MB-231 cells as well as pro-angiogenic events such as the stimulation of MMP-9 activity and VEGF expression. Together, these results demonstrate for the first time that Ang-(1-7) counteracts tumor aggressive signals stimulated by AngII in breast cancer cells emerging the peptide as a potential therapy to prevent breast cancer progression.

## INTRODUCTION

Independently of its role in homeostasis of cardiovascular and renal systems, angiotensin II (AngII) acts as a true cytokine modulating in a paracrine and autocrine manner multiple biological processes like tissue regeneration, cellular proliferation, growth factor release, and inflammation [1–3]. AngII acts through two different G protein-coupled receptors, AngII type 1 receptor (AT1) and AngII type 2 receptor (AT2), which have distinctive pharmacological and signal transducing characteristics [4]. AT1 receptor mediates most of the AngII actions, thus representing a critical pharmacological target in the treatment of several cardiovascular disorders [5, 6].

In addition to AngII, the main effector peptide of the system, other peptides like AngIII [Ang-(2-8)], AngIV [Ang-(3-8)] and Ang-(1-7) have been identified as biologically active mediators of the renin-angiotensin system (RAS). In contrast to Ang metabolites with truncation on the N-terminus (AngIII and AngIV), which still show similar detrimental effects as Ang II due to stimulation of the AT1 receptor, the heptapeptide Ang-(1-7) mainly generated by the monocarboxypeptidase angiotensin converting enzyme 2 (ACE 2), attracted special attention over the last two decades for showing the critical ability to counteract many actions of AngII in different pathophysiological settings [7–11]. Consequently, Ang-(1-7) has shown potent vasodilative, antiproliferative, antiangiogenic and antithrombotic properties [12–14]. We have previously identified the G protein–coupled receptor Mas, encoded by the Mas proto-oncogene, as a receptor being associated with Ang-(1-7)-mediated signaling [15]. Tissue RAS, where angiotensin peptides are locally produced in different organs have received special attention during the last decades [16]. Expression of RAS components such as angiotensinogen, renin, angiotensin converting enzyme, and angiotensin receptors in normal and transformed mammary ducts has been independently demonstrated by different groups [17–20]. Moreover, we described a substantial contribution of the RAS in the postlactational regression phase, where AT1 receptor signaling together with other local factors mediates apoptosis and tissue remodeling in the mammary gland [20].

It has been demonstrated that AngII plays a critical role in breast cancer development by stimulating cell proliferation of breast cancer cells and tumor angiogenesis [21–23], and modulating tumor cell migration and invasion [24–28]. In addition, it has been recently demonstrated that overexpression of AT1 receptor in breast cancer cells, induces epithelial–mesenchymal transition (EMT) and promotes tumor growth and angiogenesis [29]. EMT is a highly conserved biological process characterized by the conversion of polarized, immotile epithelial cells into mesenchymal cells with a motile phenotype. It has been shown that EMT is involved in promoting cancer cell invasion, metastasis and chemoresistance [30]. Moreover, AT1 blockers (ARBs) or ACE inhibitors have efficiently reduced tumor growth, angiogenesis, and metastasis in experimental mouse models [31–34]. However, whether other angiotensin peptides can modulate AngII pro-tumorigenic actions in mammary cells remain largely unknown. In the present work, we studied the impact of Ang-(1-7) on AngII-induced pro-oncogenic features in non-tumorigenic epithelial cells and breast cancer cells.

Here, we describe that AngII induced EMT in normal mammary epithelial cells is completely abolished by Ang-(1-7). Since migration and invasion features triggered by AngII in breast cancer cells are also blunted by the heptapeptide, it identifies Ang-(1-7) as a potential preventive therapy for breast cancer metastasis.

## RESULTS

### Ang-(1-7) inhibits ERK1/2 and AKT activation induced by AngII

Alterations on PI3K/AKT pathway have been shown to play a significant role in the development, progression, and metastatic spread of breast cancer cells. It has been shown that AngII induces AKT activation in several cell types [27, 28, 35].

**Figure 1:**
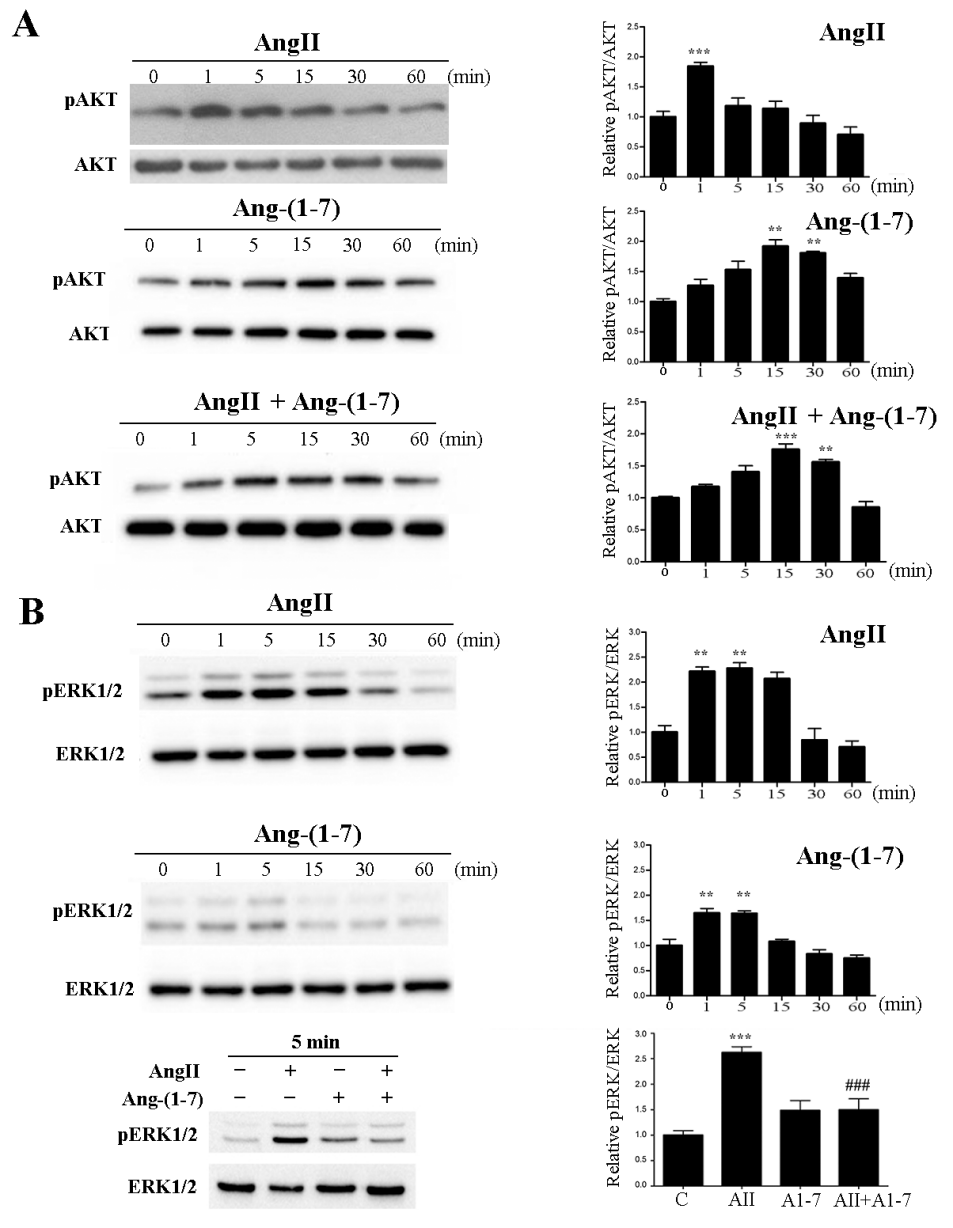
Ang-(1-7) prevents AngII-induced AKT and ERK1/2 phosphorylation in the non-tumorigenic mammary cell line NMuMG **Left panels** show representative Western blots; **right panels** show quantification by densitometry. Effect of AngII (10^-7^M) and/or Ang-(1-7) (10^-7^M) on AKT (**A**) and ERK1/2 (**B**) phosphorylation for the indicated time. WB analyses were performed using phospho-specific antibodies against ERK1/2 and AKT. The same samples were blotted with antibodies to account for total kinase expression (lower panel for each kinase). N of 3 independent experiments; Values shown in bar represent mean± SEM, **P<0.01, ***P<0.001 vs untreated cells, ^###^ P<0.001 vs AngII.

In order to study the effects of AngII and Ang-(1-7) on AKT phosphorylation, the non-tumorigenic mammary epithelial cell line NMuMG was stimulated with AngII or Ang-(1-7) at 10^-7^ M for the indicated time (Figure 1A). AngII induced AKT phosphorylation that peaked at 1 min and declined to normal at 30 min. In contrast, Ang-(1-7) induced a maximal AKT phosphorylation at 15 min (Figure 1A). When both peptides were added together, maximal phosphorylation of AKT was observed at 15 minutes, resembling the pattern of activation induced by Ang-(1-7) and disappearing the peack observed at 1 min with AngII alone. We next determined ERK1/2 phosphorylation under angiotensin stimulation (Figure 1B). While AngII strongly induced ERK1/2 activation with maximal activation at 5 min, a weaker, but significant signal was observed after Ang-(1-7) stimulation. Notably, this signal occurred earlier than the peak in AKT phosphorylation. When cells were simultaneously stimulated with both peptides, Ang-(1-7) significantly inhibited AngII induced ERK1/2 activation.

**Figure 2:**
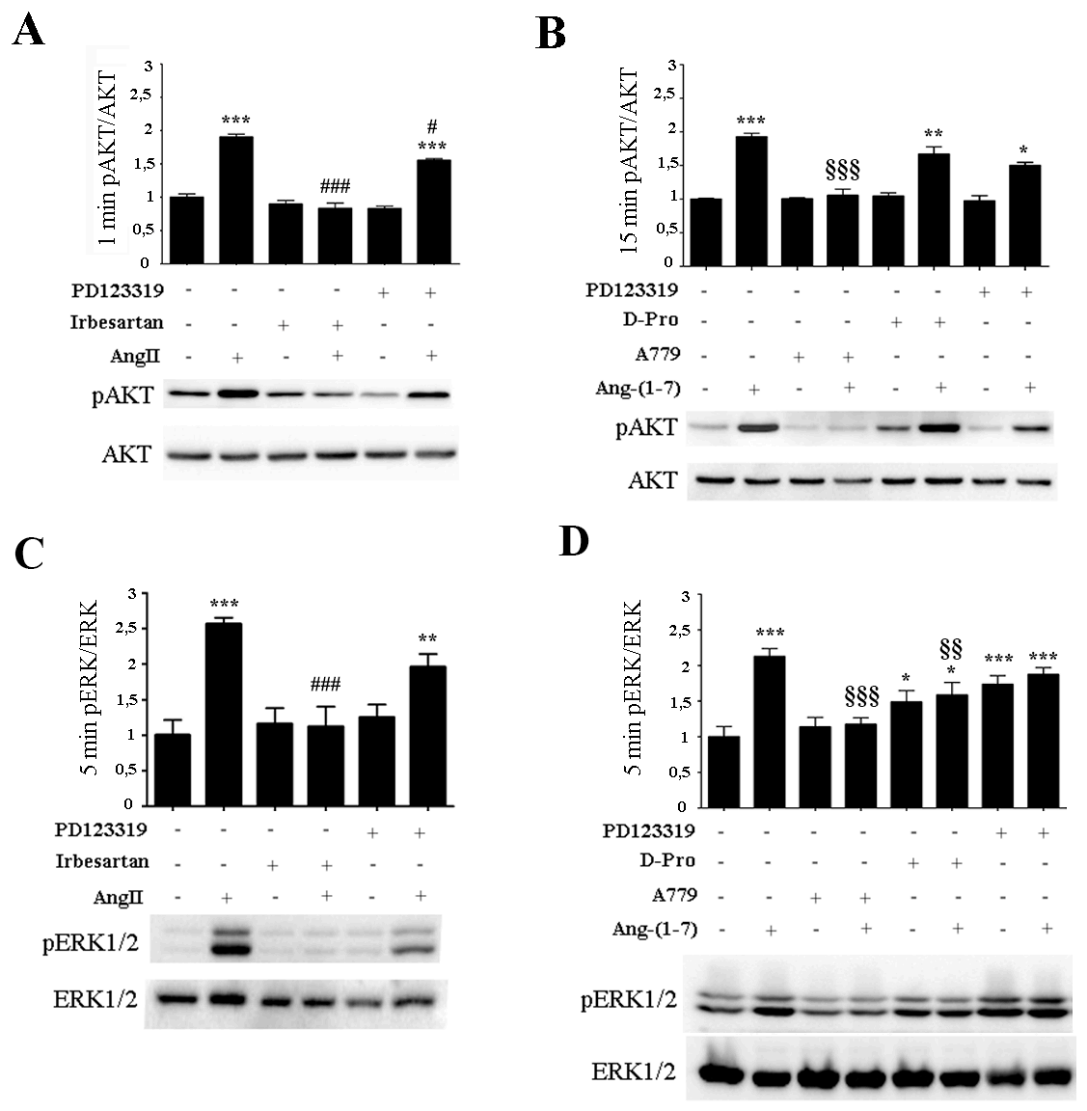
AngII induces ERK1/2 and AKT activation through AT1 receptor while Ang-(1-7) acts through the Mas receptor Western Blot analyses from the non-tumorigenic mammary cell line NMuMG were performed for p-AKT and AKT (**A-B**) and p-ERK1/2 and ERK1/2 (**C-D**). Cells were pre-incubated for 5 min with Irbesartan (10^-6^ M), PD123319 (10^-6^ M), A779 (10^-6^ M), or D-Pro (10^-6^ M), and then stimulated with AngII (10^-7^M) **(A-C)** or **(**B-D**)** Ang-(1-7) (10^-7^M) for the indicated time. Blots show representative Western blots. N of 3 independent experiments; values shown in bar represent mean±SEM quantified by densitometry and relative to control-untreated cells. *P<0.05, **P<0.01, ***P<0.001 vs untreated control cells; ^#^ P<0.05, ^##^P<0.01, ^###^ P<0.001 vs AngII or ^*§§*^P<0.01, ^*§§§*^P<0.001 *vs* Ang-(1-7) treated cells.

With the aim of identifying the receptor involved in AngII and Ang-(1-7) signal transduction, AKT or EKR1/2 phosphorylation were determined in the presence of specific pharmacological receptors blockers targeting the RAS (Figure 2). AngII alone significantly induced AKT and ERK1/2 activation compared to control levels after 1min or 5min of incubation, respectively (Figure 2A&2C). In the presence of AT2 receptor blocker PD123319, AngII maintained significant levels of both AKT and ERK1/2 activation with only a mild blockade observed. Both AKT and EKR1/2 activation induced by AngII were significantly blocked by an AT1 receptor blocker, Irbesartan, which restored values to control levels, suggesting that AngII exerts its function mainly through the AT1 receptor. In the presence of Ang-(1-7), AKT and ERK1/2 activation were induced (Figure 2A&2C). Similar levels of AKT and ERK1/2 activation induced by Ang-(1-7) were maintained in the presence of PD123319 or Irbesartan (data not shown) or the Ang-(1-7) antagonist D-Pro (D-Pro^7^-Ang-(1-7)), which has been identified as a Mas and MrgD receptor blocker in *in vitro* experiments [36, 37] (Figure 2B&2D). In contrast, treatment with the Mas receptor blocker, A779, significantly blocked AKT and EKR1/2 phosphorylation induced by Ang-(1-7) to control levels (Figure 2B&2D). Notably, ERK1/2 phosphorylation, but not AKT phosphorylation, was significantly induced with PD123319 and D-Pro alone. Thus, we cannot conclude on PD and D-Pro effects of ERK1/2 activation since the compounds stimulates ERK1/2 phosphorylation almost equally effective as Ang-(1-7) discarding significant blocking effects observed when Ang-(1-7) and D-Pro were added together (Figure 2D).

### Ang–(1-7) abolishes AngII induced epithelial-to-mesenchymal transition

**Figure 3:**
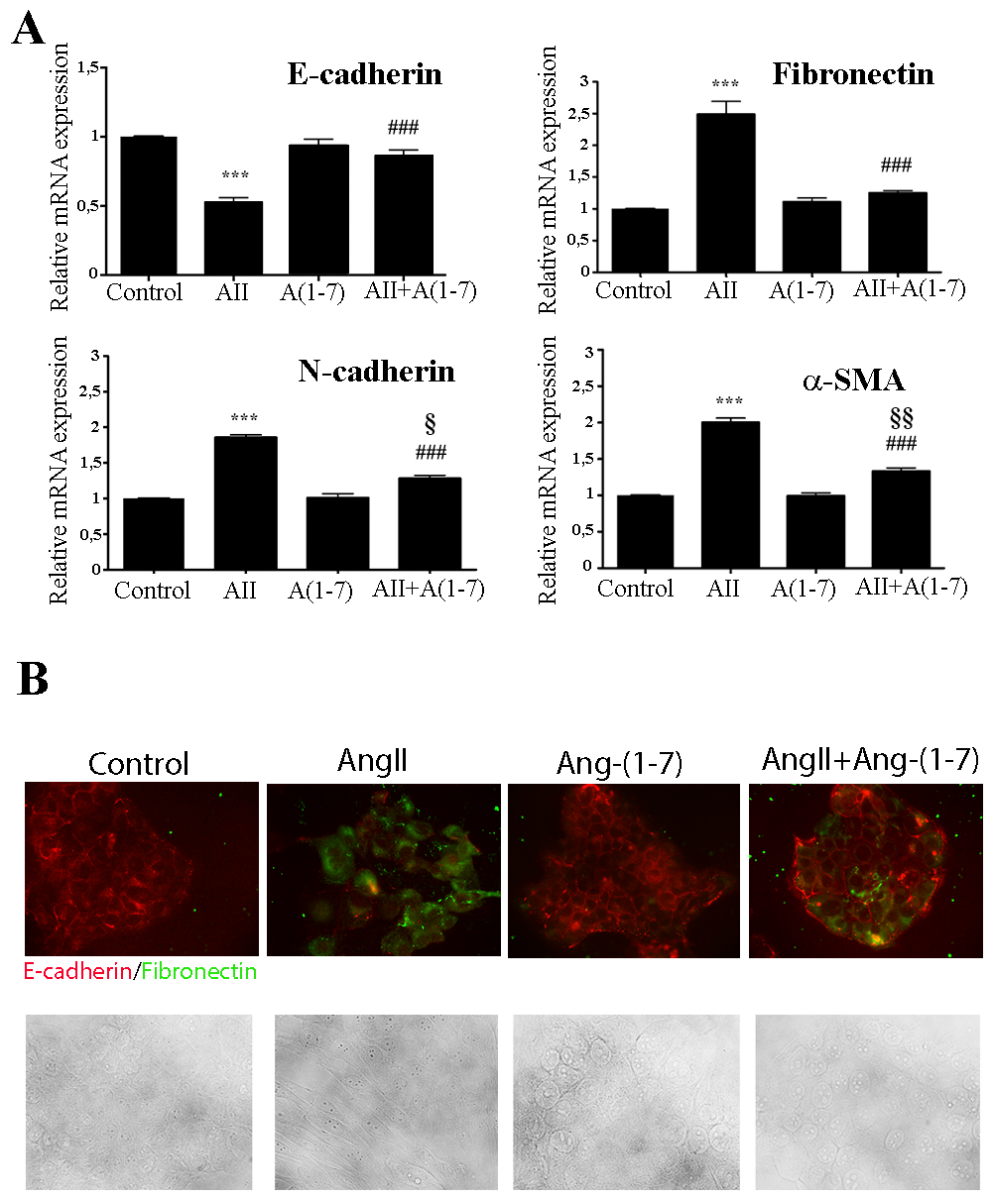
Ang-(1-7) abolish AngII-induced EMT in the non-tumorigenic mammary cell line NMuMG **(A)** Cells were treated with AngII (10^-7^M) and/or Ang-(1-7) (10^-7^M) for 3 days. The mRNA levels of E-cadherin, fibronectin, N-cadherin and ɑ-SMA were determined by qRT-PCR. mRNA levels have been normalized to GAPDH and relative to control. Bars indicate means ± SEM, n ≥ 3, ***P<0.001 *vs* control, ^**###**^P<0.001 *vs* AngII, ^*§*^P<0.05, ^*§§*^P<0.01 vs Ang-(1-7) treated cells. **(B)** Upper Panel: influence of AngII (10^-7^M) and/or Ang-(1-7) (10^-7^M) on E-cadherin and fibronectin expression and subcellular localization after 3 days of treatments. NMuMG cells were immunostained for E-Cadherin (red) or fibronectin (green). Images are shown at ×400 magnification. Lower Panel: Bright fields of morphological changes induced by AngII (10^-7^M) on NMuMG treated cells during EMT process.

Reports strongly indicate that both invasion and metastasis may be dependent on the acquisition of epithelial-to-mesenchymal transition (EMT) features by primary cancer cells [38]. During EMT, cells lose their epithelial characteristics such as cell polarity and cell-cell contact, usually measured as a decrease in E-cadherin expression, and acquires mesenchymal features such as motility and a spindle-shaped phenotype [39, 40]. These attributes increase cell motility, resulting in the release of cells from the parental epithelial tissue site and gain the ability to reconstitute metastatic colonies at distant sites. A small subpopulation of cancer cells acquires cancer stem-like cell (CSCs) traits, exhibiting mesenchymal cell features associated with increase of EMT-related markers such us N-cadherin, Vimentin, α-SMA (anti alfa-smooth muscle actin), fibronectin or Snail [41]. The non-tumorigenic mammary epithelial cell line NMuMG is a generally accepted cell model to study EMT phenomena [42]. We describe here for the first time that treatment of NMuMG cells with AngII for 3 days resulted in a transition from an epithelial to a mesenchymal phenotype (Figure 3A). In the presence of AngII, the expression of epithelial markers such as E-cadherin was inhibited (0.52 fold *vs* control) while mesenchymal markers such as fibronectin, N-cadherin and α -SMA were enhanced (fibronectin 2.49 fold, N-cadherin 1.86 fold, and α–SMA 2.0 fold) (Figure 3A). In contrast, Ang-(1-7) was unable to induce any changes on the expression of EMT markers. Importantly, when both peptides were simultaneously added to the cell culture, Ang-(1-7) abolished AngII-induced EMT changes in E-cadherin and fibronectin and partially blocked the changes in N-cadherin and α–SMA (Figure 3A). Similar results and morphological changes were observed when immunofluorescence was performed to evaluate EMT markers on these cells (Figure 3B), with Ang-(1-7) preventing not only suppression of E-cadherin but also upregulation of fibronectin stimulated by AngII.

### AngII induces EMT and migration through AKT activation

**Figure 4:**
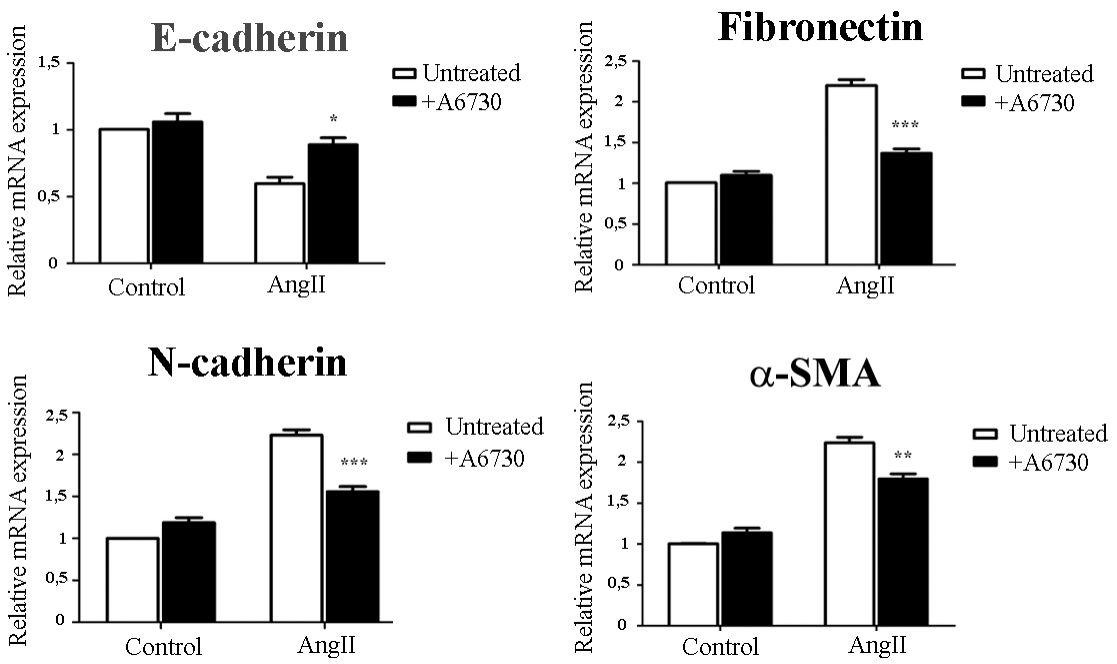
AngII induces EMT and through AKT activation NMuMG cells were treated for 3 days with AngII (10^-7^M) in the presence or absence of an AKT inhibitor A6730 (1uM). The mRNA levels of E-Cadherin, Fibronectin, N-cadherin and ɑ-SMA were determined by qRT-PCR. mRNA levels have been normalized to GPDH and relative to control. Bars indicate means ± SEM, n ≥ 3, *P<0.05, **P<0.01, ***P<0.001 *vs* untreated cells.

We further examined whether AKT activation is involved in AngII-induced EMT in mammary epithelial cells. We found that in the presence of the an AKT1/2 kinase inhibitor, A6730, the expression of the epithelial and mesenchymal EMT biomarkers were almost restored to control levels, with highest efficacy on E-cadherin and fibronectin expression (Figure 4). These results suggest that AKT phosphorylation is at least in part necessary for AngII-induced EMT.

### Ang-(1-7) prevents AngII-induced metastatic features on breast cancer cells

**Figure 5:**
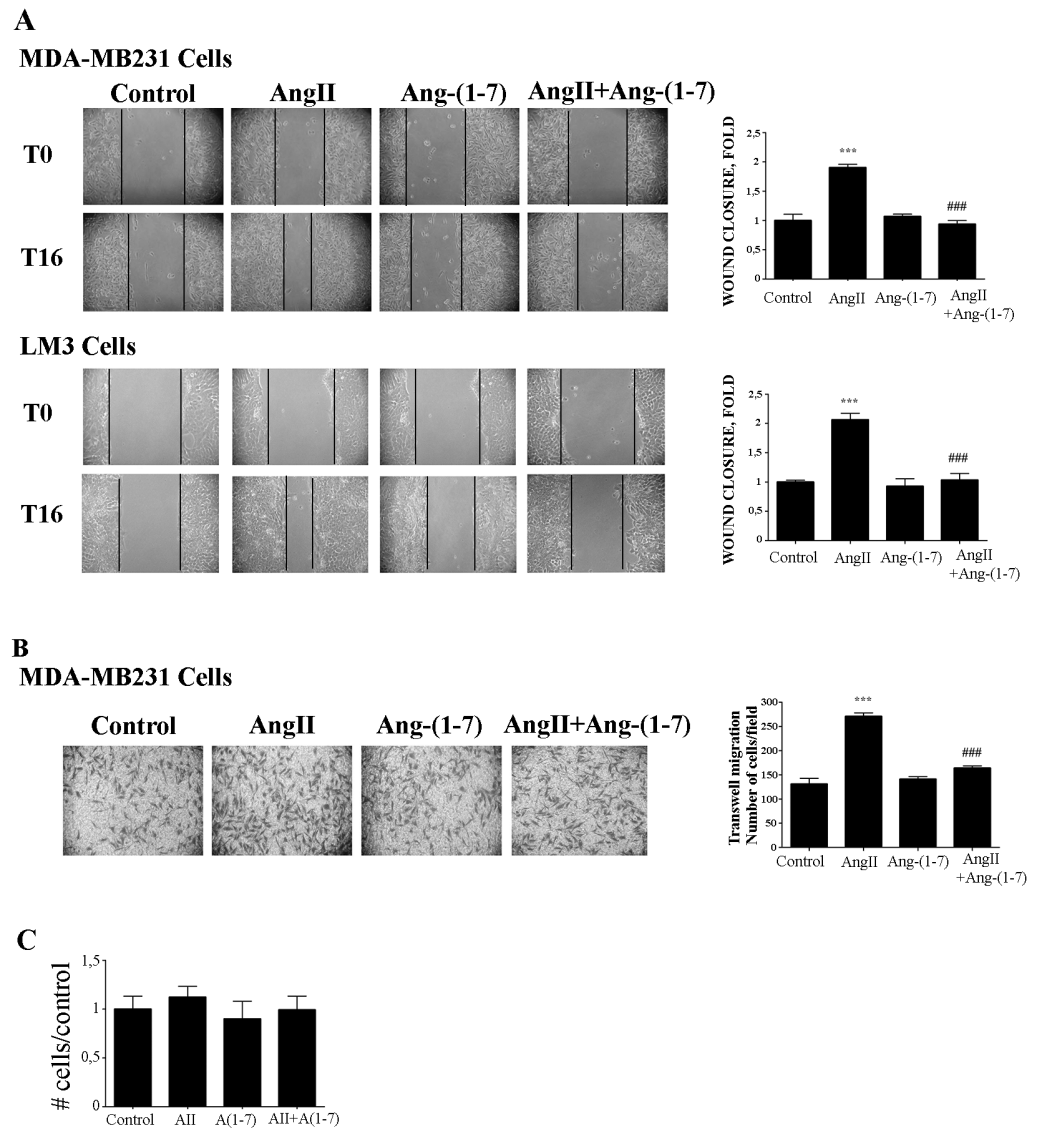
Ang-(1-7) blockes AngII induced tumor cell migration **(A)** Wound healing assay on MDA-MB231 or LM3 cells. Wounds were registered by phase contrast microscopy immediately after scratching (T0) and after 16 h in serum-free medium (T16) treated with AngII (10^-7^M) and/or Ang-(1-7) (10^-7^M). Bars show results from 3 independent experiments performed and expressed as fold increase of wound closure at time 16 hrs (T16) compared to control (untreated cells). Representative pictures of wounds at T0 and T16, magnification, 100x. Bars indicate means ± SEM, ***P <0.001 vs control, ^**###**^*P*<0.001 vs AngII. **(B)** Transwell cell migration assayed with MDA-MB231 cells in serum-free media across 8 mm-pore filters coated with type I collagen. The cells that migrated to the lower portion of the chamber, were fixed and stained with cristal violet, and five fields per well were counted. Bars indicate number of migrated cells, means ± SEM, n ≥ 3 ***P <0.001 *vs* control or ^**###**^P<0.001 *vs* AngII. **(C)** AngII (10^-7^M) and Ang-(1-7) (10^-7^M) had no effect on cell proliferation measured over 24 h by cell counting and MTS assay in MDA-MB231 cells.

We next evaluated the effects of AngII and Ang-(1-7) on cell migration and invasion in two potentially metastatic mammary cancer cells lines: MDA-MB-231 (human) and LM3 (mouse). We found that Ang-(1-7) completely blocked AngII-induced tumor cell migration either performed on wound healing or in transwell assays (Figure 5A-5B). The migration observed was not due to proliferation, since neither AngII (10^-7^M) nor Ang-(1-7) (10^-7^M) did induce cell proliferation after 24h of treatment (Figure 5C).

**Figure 6:**
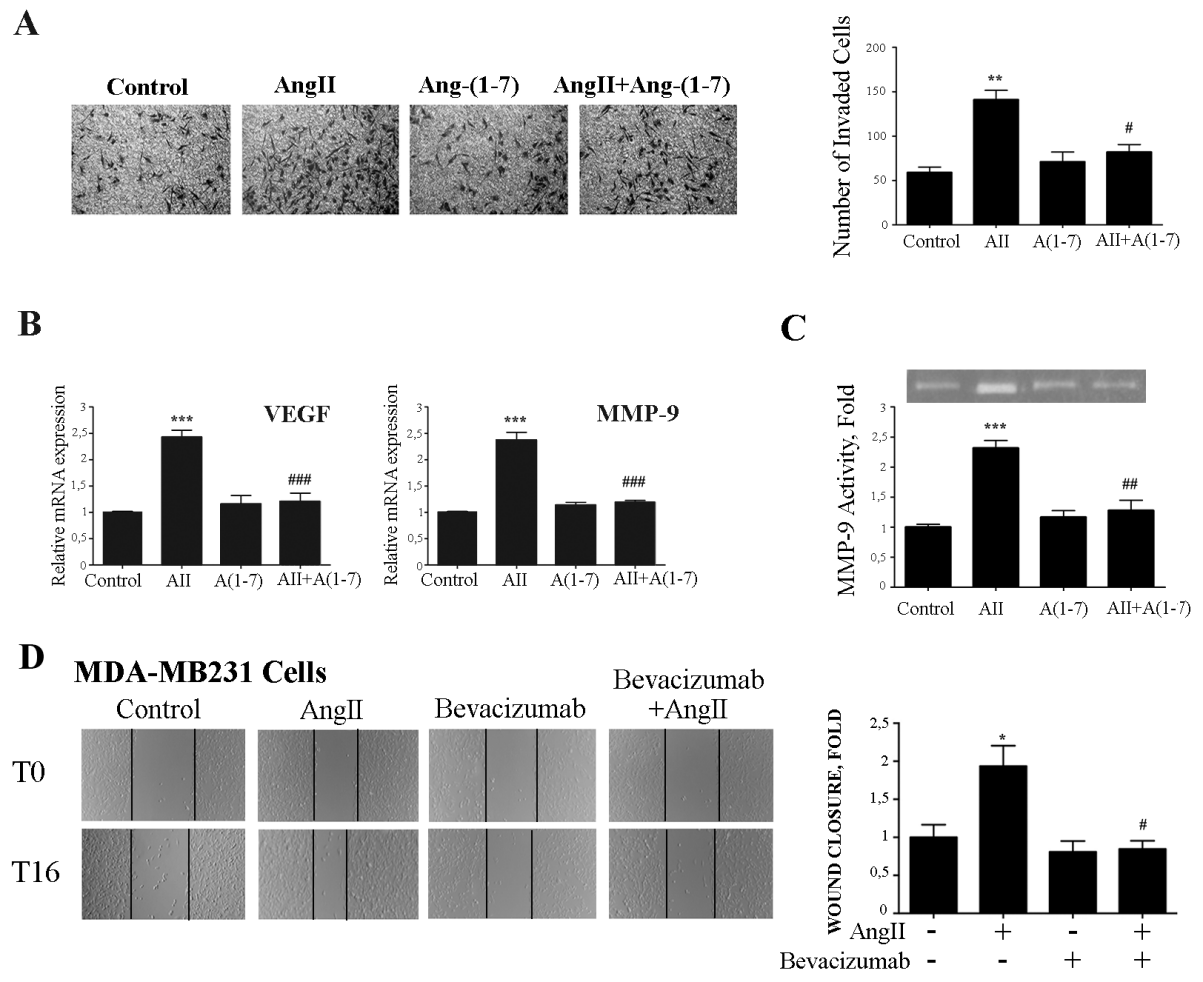
Ang-(1-7) abolishes AngII-induced breast cancer cell invasion, MMP-9 activity and VEGF and MMP-9 expression **(A)** Transwell cell invasion assayed of MDA-MB231 cell in serum-free media across 8 mm-pore filters coated with matrigel. Cells that migrated to the lower portion of the chamber were fixed and stained with cristal violet, and five fields per well were counted. Bars indicate number of invaded cells, means ± SEM, n ≥ 3, **P <0.01 *vs* control or ^**#**^*P*<0.05 *vs* AngII. **(B)** VEGF and MMP-9 mRNA expression levels were determined by qRT-PCR on MDA-MB231 cells treated with AngII (10^-7^M) and/or Ang-(1-7) (10^-7^M) for 24 h. mRNA levels have been normalized to GAPDH and relative to control. Bars indicate means ± SEM, n ≥ 3 ***P <0.001 *vs* control or ^**###**^P <0.001 *vs* AngII. **(C)** Gelatin-based zymography analysis of MMP-xs9 activity in conditioned medium of MDA-MB231 cells treated as in B. Image shown is one representative of 3 independent experiments (Upper panel). For quantification (ImageJ software), results were normalized to the quantity of proteins in cell lysate and expressed relative to control (lower panel). Bars indicate means ± SEM, n ≥ 3, ***P <0.001 vs control or ^**##**^*P* <0.01 vs AngII. **(D)** Wound healing assay on MDA-MB231 cells. Wounds were registered by phase contrast microscopy immediately after scratching (T0) and after 16 h in serum-free medium (T16) treated with AngII (10^-7^M) and/or bevacizumab (100 ug/ml). Bars show results from 3 independent experiments performed and expressed as fold increase of wound closure at time 16 hrs (T16) compared to control (untreated cells). Representative pictures of wounds at T0 and T16, magnification 100x. Bars indicate means ± SEM, *P <0.05 vs control, ^**#**^*P*<0.05 vs AngII.

Similar results were obtained in invasion assays using filters coated with matrigel that mimics the extracellular matrix. As shown in Figure 6A, Ang-(1-7) also abolished the invasion induced by AngII on breast cancer cells.

Vascular endothelial growth factor (VEGF) has been identified as a potent cytokine involved in tumor angiogenesis and metastasis formation [43]. When breast cancer cells were stimulated with AngII, the expression of VEGF was increased (2.39 fold). Interestingly, cotreatment of Ang-(1-7) with AngII completely abolished this increase in VEGF mRNA levels in breast cancer cells (Figure 6B). Similar to Ang-(1-7), the treatment with an anti-VEGF antibody (bevacizumab) abolished AngII –induced cell migration of MDA-MB-231 cells (Figure 6D).

Tumor cells are believed to utilize the matrix metalloproteases degrading capability to spread to distant sites. Previous studies have extensively documented AngII-mediated activation of MMP-9 in several cell types [44]. In agreement with those studies, we have previously demonstrated that AngII, through AT1 receptor, activates MMP-9 activity during mammary gland involution [20]. As depicted in Figure 6B-6C, Ang-(1-7) abolished MMP-9 expression and activity triggered by AngII on breast cancer cell line MDA-MB231.

## DISCUSSION

Our study shows that Ang-(1-7) has the ability to counteract AngII-induced metastatic features in breast cancer cells. Pro-metastatic effects of AngII in various experimental models *in vivo* and *in vitro* have been well documented and attributed to its actions on the host microenvironment [31, 32, 45, 46]. Our study confirms the reported stimulating effects of AngII on breast cancer cell migration, invasion and activation of pro-metastatic factors such as MMP-9 and VEGF, supporting the notion that AngII can promote tumor cell growth and metastasis progression. We show here for the first time that Ang-(1-7), acting mainly through the Mas receptor, abolishes AngII-induced migration, invasion, VEGF expression, and MMP-9 activity in breast cancer cells. Moreover, we found that Ang-(1-7) completely blockes AngII-induced EMT, which is an essential step occurring during metastasis progression.

Dysregulation of the PI3K/AKT pathway occurs in more than 70% of breast cancers [47]. PI3K can be activated by RTKs or downstream of GPCRs through direct interaction with heterotrimeric G protein subunits [48]. In the specific setting of post-lactational regression phase, we have previously observed AKT and ERK1/2 pathway activation in the presence of the AT1 receptor blocker Losartan in an *in vivo* model [20]. Previous findings support a dose-dependent effect of AngII on cancer cell proliferation and cell survival through the PI3K/Akt pathway [27, 28, 44]. In addition, AngII-mediated cell migration in choriocarcinoma cells can be abolished by a selective AT1 antagonist and a PI3K inhibitor [49]. In MDA-MB-231 cells, AngII induced AKT activation, and pretreatment of cells with PI3K or AKT inhibitors significantly reduced AngII-mediated cell migration and MMP-2 and-9 upregulation [44]. In agreement with these results, we found that AngII induced AKT phosphorylation in mammary epithelial cells at a very early time point (1 min). In contrast, Ang-(1-7) induced AKT phosphorilation at a later time point (15 min). When both peptides were added together, the pattern of activation resembled that of Ang(1-7) blunting the early effect of AngII. Moreover, to identify the receptors responsible for AngII and Ang-(1-7) mediated AKT phosphoryation, we used receptor-type-specific blockers. We found that AT1 and Mas receptor, were involved in AngII and Ang-(1-7)-mediated AKT phosphorylation, respectively. Furthermore, we could demonstrate in this study that AngII-induced EMT in non-tumorigenic mammary epithelial cell line NMuMG requires AKT activation.

Overexpression of AT1 in breast cancer cells induces EMT and promotes tumor growth and angiogenesis. AT1-overexpressing cells exhibited a mesenchymal-like phenotype, together with an increase in nuclear accumulation of phospho-Smad3 and Snail, increased Smad4 and N-cadherin levels, and a loss in E-cadherin [29]. Reduction in Smad4 has been shown to suppress TGF-β-induced responses associated with EMT in mammary epithelial NMuMG cells *in vitro* [50]. Furthermore, recent studies have reported that AT1 stimulation by AngII induces EMT via the Smad signaling pathway in renal epithelial cells and vascular smooth muscle cells *in vitro* [51, 52]. EMT is often associated with aggressive, invasive phenotypes and malignant tumor progression [42]. We show here for the first time that EMT induced by AngII in mammary epithelial cells is partially blocked by Ang-(1-7). Interestingly, the concomitant treatment with both angiotensin peptides, restored E-cadherin expression. This finding suggests that Ang-(1-7) promotes attachment of epithelial cells to the extracellular matrix becoming less prone to migrate.

Ang-(1-7) is a natural bioactive peptide of the renin-angiotensin system produced in many tissues by ACE2 using AngII as a substrate. It has been shown that Ang-(1-7) reduces the growth of human lung tumor xenografts, with a concomitant decrease in VEGF and reduced vessel density, as well as decreasing the growth of orthotopic human estrogen receptor–positive or HER2-overexpressing breast tumor xenografts [10, 53, 54]. It has also been recently demonstrated that Ang-(1-7) decreases cell growth and angiogenesis of human nasopharyngeal carcinoma xenografts [55]. However, there are only very few reports on Ang-(1-7)’s role in breast cancer development. We demonstrate here that in a highly aggressive and metastatic breast cancer cell line Ang-(1-7) completely prevented AngII-induced cell migration and invasion.

Besides, our study shows that AngII-induced ERK1/2 activation is inhibited by Ang-(1-7). Several studies have shown that activation of ERK1/2 plays an important role in cell migration of several tumors [56]. Our studies are also in agreement with the previous studies demonstrating that Ang-(1-7) decreases the ERK1/2 signal transduction pathway in lung and prostate cancer [14, 53]. It is likely that Ang-(1-7) activates a signaling pathway that negatively regulates AngII-induced ERK1/2 and AKT activation and through this mechanism is able to block breast cancer migration and invasion induced by AngII. Further studies unveiling the exact mechanism through which Ang-(1-7) counteracts AngII effects might help to provide a better understanding of their role in breast cancer development.

Enzymatic degradation of ECM is one of the crucial steps in cancer invasion and metastasis. MMP-9, is the main extracellular matrix protein-degrading enzyme known to play an important role in breast cancer cell migration and invasion [57, 58]. In breast tumor D3H2LN cells, AngII upregulates MMP-2 / MMP-9 and ICAM1, which are involved in cell adhesion, migration, and invasion [59]. In MDA-MB-231 cells, specific blocking of MMP-2 and MMP-9 by siRNA significantly suppressed AngII-induced cell migration [24, 44]. In this study, we found that the enhanced expression and enzymatic activity of MMP-9 induced by AngII was significantly abolished by Ang-(1-7). Moreover, we also found that Ang-(1-7) in breast cancer cells also inhibits AngII-induced VEGF expression, a robust stimulator of angiogenesis. Notably, VEGF expression is required for the increased tumor initiation capacity of breast cancer cells that have undergone EMT [43]. These results are in agreement with previous studies showing significant VEGF reduction in nasopharyngeal carcinoma cell lines overexpressing Ang-(1-7) [55]. As we also show in this study that anti-VEGF treatment of triple negative breast cancer cells completely suppresses AngII-induced migration, our results may also indicate the potency of a new strategy, using a combined treatment of Ang-(1-7) and anti- VEGF to prevent invasion, angiogenesis, and metastasis of aggressive breast cancer tumors.

The opposing biological effects of Ang-(1-7) and AngII in different scenarios has been vastly demonstrated [60]. However, whether Ang-(1-7) had any protective effect against breast cancer progression remained unknown. In the present study, we explored this possibility and we found that Ang-(1-7) reverses EMT, migration, invasion, VEGF, and MMP-9 activity induced by AngII. Consequently, in our experimental settings, activation of the Ang-(1-7)/Mas receptor axis acts as a physiological antagonist of the AngII/AT1 receptor axis. Therefore, it is worth to evaluate in upcoming experiments, whether Ang(1-7) represents a better alternative to prevent breast cancer progression than the treatment with an AT1 receptor blocker.

## MATERIALS AND METHODS

### Reagents and antibodies

D-Ala^7^-Ang-(1-7) (A779), Irbesartan, AngII and Ang-(1-7) were purchased from Bachem AG, Bubendorf, Switzerland, Losartan from Sigma-Aldrich (St. Louis, MO, USA), and PD123319 (PD) from Parke-Davis Pharmaceutical Research (Detroit, MI, USA). D-Pro^7^-Ang-(1-7) (D-Pro) was manufactured by Biosyntan (Berlin, Germany), the monoclonal anti-VEGF antibody, bevacizumab, was purchased from Roche, Switzerland), antibodies against AKT and pAKT from Cell Signaling (Beverly, CA, USA) and against ERK and pERK from Santa Cruz Biotechnology Inc. (Santa Cruz CA, USA.). The primary antibody for immunofluorescence, anti-E-cadherin/CDH1 (ECCD-2), was purchased from Zymed – Thermo Scientific (Massachusetts, USA), and anti-fibronectin from Gibco-BRL (Massachusetts, USA). The secondary antibodies for immunofluorescence, anti-rat Alexa Fluor 568 and anti-rabbit Alexa Fluor 488, came from Thermo – Scientific. The other secondary antibodies used including horseradish peroxidase (HRP)-conjugated anti-rabbit and mouse IgG have been delivered by Santa Cruz Biotechnology Inc. AKT inhibitor A6730 (Akt1/2 kinase inhibitor), phosphatase inhibitor, and protease inhibitor cocktail were purchased from Sigma. Oligos for qRT-PCR were acquired from Invitrogen (Carlsbad, CA, USA).

### Cell culture and treatments

NMuMG cells were grown in Dulbecco’s Modified Eagle’s Medium (DMEM high glucose, Invitrogen (Massachusetts, USA) supplementary with 10% fetal bovine serum; MDA-MB231 cells were grown in RPMI 1640 supplementary with 10% fetal bovine serum; and LM3 cells, derived from mammary adenocarcinoma spontaneously occuring in Balb/c mice, were gently provided by Dr. Elisa Bal de Kier Joffé, (Roffo Institute, Buenos Aires, Argentina) and grown in MEM supplementary with 5% fetal bovine serum. All cells were incubated at 37°C in a humidified atmosphere of 5% CO_2_. For Wound Healing Migration assays, MDA-MB231 and LM3 cells were grown to confluence and then starved in serum-free medium during 8 h before being treated with AngII, Ang-(1-7), or both (10^-7^M). For transwell migration and invasion assays, cells were starved for 24 in medium supplemented with 1% serum and then incubated with AngII, Ang-(1-7) or both 10^-6^ M in serum free medium. For CellTiter 96* Aqueous One Solution Cell Proliferation Assay (MTS), zymography, and MMP-9 and VEGF expression assays, the AngII/Ang-(1-7) concentration was 10^-7^ M. For Western blot (WB) assays, NMuMG cells were starved in serum-free medium for 3 h, and then, cells were stimulated at different time points with AngII, Ang-(1-7), or both at a final concentration of 10^-7^ M. When treated with AT1, AT2, and Mas receptor blockers, cells were pre-incubated for 5 min with the corresponding antagonists, at a final concentration of 10^-6^ M. For EMT assays, cells were starved over-night in 2% serum-supplemented medium, and then treated with AngII, Ang-(1-7), or both in a concentration of 10^-7^ M, every 24 h for 3 days. Cells stimulated with TGF-β (4 ng/ml; positive control), received treatment every 48 h for 3 days. In assays in which the AKT inhibitor, A6730, was used, its final concentration was 1 μM, and cells were pre-incubated with it for 1 h.

### Quantitative RT-PCR

Quantitative Real-Time PCR was performed as previously described [61]. cDNA was synthesized from 1 μg of total RNA using oligo-dT and reverse transcriptase (Reverse Transcriptase MMLV, Promega, Madison, USA) as recommended by the manufacturer. PCR amplification (35 cycles) was performed on 20 ng cDNA using oligonucleotide primers as follows:

- Mouse E-cadherin forward GCTTCAGTTCCGAGGTCTACAC, reverse CTGTGATGGTGCCGTCTGTC;
- Mouse fibronectin forward TACCAAGGTCAATCCACACCCC, reverse CAGATGGCAAAAGAAAGCAGAGG;
- Mouse GAPDH forward GAGTCAACGGATTTGGTC, reverse CGAAGGTGGAAGAGTGGGAGTTG;
- Human GAPDH- forward GAGTCAACGGATTTGGTC, reverse TTGATTTTGGAGGGATCTCG;
- Mouse MMP9 forward AGACCTGGGCAGATTCCAAACC, reverse GCAAAGGCGTCGTCAATCACC;
- Mouse N-cadherin forward TGGATGAAACGGCGGGATAA, reverse TGTGGCTCAGCATGGATAGG;
- Mouse α-SMA forward ACCACCATGTACCCAGGCATT, reverse GCTGGAAGGTAGACAGCGAAG;
- Mouse VEGF-F forward GCCCACTGAGGAGTCCAACA, reverse GCTGGCCTTGGTGAGGTTT.

### Western blot analysis

Cells were solubilized in lysis buffer (30 mM NaCl, 0.5% TritonX-100, 50 mM Tris–HCl; pH7.4) containing a cocktail of phosphatase and protease inhibitors. Supernatant was collected (14,000 ×g, 4°C, 20 min), and protein concentration was determined with a Bradford protein assay kit (Bio-Rad Laboratories, California, USA). Equal quantities of protein (25 μg) were subjected to SDS-PAGE and transferred onto PVDF membranes (GE Healthcare Life Science, Buckinghamshire, UK). The membranes were incubated at 4°C overnight with primary antibodies ERK1/2 (1:1,000), p-ERK1/2 (1:1,000), AKT (1:1,000), pAKT (1:1,000), GAPDH (1:1,000), diluted in 3% of dry low-fat milk or 3% BSA for phosphorylated antibodies, followed by incubation with horseradish peroxidase (HRP)-conjugated rabbit or mouse IgG (1:5,000). Signal intensity was detected using an Enhanced Chemiluminescence Kit (ECL Prime, Amersham, GE Healthcare, Buckinghamshire, England) and quantified using Syngene G-Box XR5 (Syngene, Maryland, USA).

### Zymography

Conditioned medium from MDA-MB-231 cells, previously treated for 24 h with AngII, Ang-(1-7), or both in serum-free medium, were collected and centrifuged at 800 rpm for 5 min to remove cellular debris. Samples were then subjected to electrophoresis on gelatin substrate gels (8.8% SDS-polyacrylamide slab gels containing 1 mg/ml gelatin). MMP9 activity was visualized as a clear band at 90 kDa after Coomassie Blue coloration. The zymograms were scanned and subjected to densitometric analyses using the PC version of NIH Image J (Scion Corp., Frederick, MD, USA).

### Immunofluorescence

NMuMG cells were grown on sterile glass cover slips in 12-well plates for 4 days and immediately fixed with 4% Paraformaldehyde in PBS. After being washed 2x with PBS, they were incubated for 15 min with a PBS + 0.1% Triton X-100 solution, and then blocked 1 h with 5% BSA in PBS + Triton X-100 0.025% solution. Then, they were washed and incubated with the appropriate primary antibodies over-night at 4°C: anti E-cadherin (1:2,000) and anti-fibronectin (1:100) diluted in a 3% BSA/PBS-T solution, or vehicle as an isotype control. Cells were incubated with specific secondary antibodies: Alexa 568 conjugated goat ant-rat or Alexa 488 conjugated goat ant-rabbit, diluted (both 1:400) in 3% BSA/PBS-T solution. Nuclei were counterstained with 4′,6-diamidino-2-fenylindole (DAPI; Sigma Aldrich, St. Louis, MO, USA) for 10 min. Sections were finally air-dried and mounted with 80% glycerol. Immunofluorescence pictures were acquired with Olympus IX-81 fluorescence microscope (Tokyo, Japan). ImageJ was used for processing and analysis of the signal intensity.

### Migration Assays

For wound healing assays with MDA-MB231 or LM3 cells, 500,000 cells were grown to confluence in 6-well plates and, after starvation, cross-shape wounds were performed in the monolayer using a sterile 10-μl pipette tip. Wounds were scanned by phase contrast microscopy immediately after scratching (T0) and after 16 h in serum-free medium (T16) treated with AngII (10^-7^M) and/or Ang-(1-7) (10^-7^M) and or bevacizumab (100 ug/ml). For wound-closure area quantification, 3 images per wound were captured, in a total of 2 wounds per well, at T0 and T16, using a camera (Nikon Coolpix P5100; Tokyo, Japan) integrated to a white field inverted microscope (Nikon Eclipse TS100). The areas were quantified using Image Pro-Plus software (Media Cybernetics, Inc. Rockville, MD, USA). For each condition, the wound closure was calculated as the ratio of wound diameter at T16 relative to T0.

For transwell migration assays, cell motility was tested in 8-μm pore polycarbonate membrane transwell chambers (Corning Inc, NY, USA). Membranes were coated on both sides with 25 μg/ml of rat tail collagen I (Roche, Basel, Switzerland). Cells were starved in medium (1% serum) for 24 h. For plating in transwells, cells were re-suspended in serum-free medium and 80,000 cells/250 μl were added to the top chamber, with the corresponding angiotensin or PBS as negative control. Five hundred μl serum-free medium was added to the bottom chamber, except for one which contained 10% serum as a positive control of migration. Cells were allowed to migrate for 20 h. For quantification, non-migrated cells were scraped from the top membrane, and migrated cells in the lower chamber were fixed in fresh 4% PFA for 20 min and stained in 0.1% crystal violet for 30 min. Once washed with H_2_O and air dried, pictures were taken, and migrated cells were counted in five different random fields in duplicate wells using a camera integrated to a white field inverted microscope. The number of cells/field was counted using the ImageJ 1.37v software, and results were expressed as the number of treated cells capable to migrate per field. The results were expressed showing the mean ± SEM.

### Invasion Assay

Invasion was tested in 8-μm pore polycarbonate membrane transwell chambers coated with matrigel matrix 0.3 mg/ml (Corning, NY, USA) according to the manufacturer’s recommendations. For plating in transwells, cells were re-suspended in serum-free medium, and 1.5 × 10^5^ cells/250 μl were added to the top chamber with the corresponding angiotensin or PBS as a negative control. Five hundred μl serum-free medium was added to the bottom chamber, except for one well which contained 10% serum as a positive control of invasion. Cells were allowed to migrate for 24 h and then proceeded following the transwell migration assayed describe above.

### Cell viability assay

Cell viability was assessed using the CellTiter 96* Aqueous One Solution Cell Proliferation Assay [MTS, 3-(4,5-dimethylthiazol-2yl)-5-(3-carboxymethoxyphenyl)-2-(4-sulfophenyl)-2H-tetrazolium] (Promega, Madison, WI, USA). Three thousand MDA-MB231 cells were grown in a 96-well plate and starved in medium containing 2% FBS. After 24 h, cells were stimulated with AngII, Ang-(1-7) or both (10^-7^ M) in serum-free medium. The cell proliferation assay was then performed according to the manufacturer’s instructions. The quantity of formazan product was determined by measuring absorbance at 450 nm using a microplate sepectophotometer (Glomax Multi Detection System, Promega, WI, USA).

### Statistical Analysis

Statistical significance of differences was determined with One-way ANOVA followed by Tukey posttest. Data are as mean ± SEM. A *P* value of less than 0.05 was considered statistically significant.
